# Comparison of Clinical Outcomes Between Calfactant and Poractant-Alfa in Preterm Infants with Respiratory Distress Syndrome

**DOI:** 10.3390/children12101350

**Published:** 2025-10-08

**Authors:** Leyla Sero, Nilufer Okur, Duygu Tuncel

**Affiliations:** 1 Clinics of Pediatrics, Gazi Yasargil Training and Research Hospital, Diyarbakır 21090, Turkey; 2 Clinics of Neonatology, Gazi Yasargil Training and Research Hospital, Diyarbakır 21090, Turkey

**Keywords:** prematurity respiratory distress syndrome, poractantalfa, calfactant

## Abstract

**Highlights:**

**What are the main findings?**

- Poractant alfa was associated with higher initial dosing, more frequent use of less invasive administration techniques (LISA/INSURE) and lower cost compared with calfactant.
- In infants treated with poractant alfa, the need for a second surfactant dose was significantly reduced.

**What is the implication of the main finding?**

- Both surfactant preparations provide comparable efficacy for short- and mid-term outcomes, supporting their use in neonatal RDS management.
- Practical advantages of poractant alfa—such as lower cost and better feasibility for LISA/INSURE—may guide clinical and pharmacoeconomic decision-making in NICUs.

**Abstract:**

Background: This study compares short- and mid-term morbidity and mortality outcomes in preterm infants treated with the natural surfactants poractant alfa and calfactant, to assess differences in their clinical efficacy and safety profiles. Methods: In this prospective cohort study, preterm infants (25 ^0⁄7^–32 ^6⁄7^ weeks gestation) admitted to a Level III NICU between January 2023 and March 2024 received either poractant alfa or calfactant according to hospital supply. The primary outcome was moderate-to-severe bronchopulmonary dysplasia (BPD) and/or mortality. Secondary outcomes included extubation success, need for repeat surfactant dosing, and treatment cost per patient. Short- and mid-term morbidity and mortality outcomes were compared between groups. Results: The study included 215 preterm infants (137 in the poractant alfa group; 78 in the calfactant group). Use of less invasive surfactant administration (LISA) and INSURE techniques was significantly higher in the poractant alfa group (32.8% vs. 10.3%; *p* < 0.01). Poractant alfa was administered earlier, at higher per-kg doses, and at lower median treatment cost. Rates of moderate-to-severe BPD, the composite outcome BPD or mortality, reintubation, duration of respiratory support, and length of hospitalization did not differ significantly between groups. In the poractant alfa group, the need for a second surfactant dose was lower (*p* = 0.027). Overall mortality was similar (21.2% vs. 24.4%; *p* = 0.13), with no significant difference in timing of death. Conclusions: Compared with calfactant, poractant alfa offers practical advantages—such as a higher initial dose, lower cost, and reduced need for a second repeat dose—while yielding comparable short- and mid-term morbidity and mortality outcomes.

## 1. Introduction

Respiratory distress syndrome (RDS) remains a leading cause of morbidity and mortality among preterm infants, with its incidence inversely correlated with gestational age and birth weight [1]. To mitigate the adverse effects associated with RDS, several evidence-based interventions—such as antenatal corticosteroid administration, early initiation of continuous positive airway pressure (CPAP), and timely surfactant therapy—are widely recommended [2]. Since its initial discovery in the 1950s, surfactant therapy has become a cornerstone in the management of neonatal RDS, particularly from the 1990s onward. Clinical evidence consistently shows that surfactant administration significantly reduces neonatal mortality, shortens the duration of mechanical ventilation, and decreases the incidence of air-leak syndromes, bronchopulmonary dysplasia (BPD), and BPD or death [2]. Although surfactant therapy incurs substantial costs, its proven benefits in decreasing mortality and long-term complications have established it as a globally accepted and indispensable intervention in neonatal intensive care. Despite advancements in perinatal and neonatal care, RDS continues to account for a considerable proportion of preterm infant deaths, underscoring the critical importance of optimal surfactant selection and administration strategies in postnatal management.

Natural surfactants are preferred in the treatment of RDS due to their well-established efficacy and safety profiles. Numerous comparative studies have evaluated different natural surfactant preparations, consistently demonstrating their beneficial effects on respiratory function and their role in reducing RDS-associated mortality. According to European guidelines for the management of RDS, administration of poractant alfa at an initial dose of 200 mg/kg is associated with improved survival outcomes [2]. Poractant alfa is widely utilized due to its high phospholipid content and cost-effectiveness, which are attributed to its lower required dosing volume.

Comparative analyses of animal-derived natural surfactants have shown that poractant alfa provides comparable or superior clinical outcomes to calfactant, with fewer complications and at lower doses [3]. Furthermore, studies examining the three most commonly used animal-derived surfactants have reported that calfactant achieves clinical outcomes similar to those of poractant alfa, including the duration of mechanical ventilation. However, some evidence suggests that poractant alfa may be more effective in reducing the need for repeated dosing and in lowering mortality rates compared with other surfactant formulations [4,5,6,7].

In recent years, comparative studies assessing the efficacy of natural surfactant preparations for the treatment of RDS in preterm infants have gained increasing attention, particularly with the decline in beractant use and emerging evidence favoring poractant alfa. Systematic reviews and large retrospective database analyses indicate that poractant alfa, compared with beractant, may reduce mortality and the need for repeat dosing, and in some studies has been associated with lower mortality and shorter duration of mechanical ventilation compared with calfactant. Conversely, several multicenter observational studies have found no significant differences among the three surfactants in terms of mortality, BPD, or air-leak syndromes, with some outcomes even favoring calfactant. Cost-effectiveness evaluations have further suggested that poractant alfa may be substantially more expensive than calfactant without consistently reducing the need for repeat dosing [4,5,6,7].

The primary objective of this study was to compare the efficacy of two commonly used natural surfactant preparations—calfactant and poractant alfa—in the treatment of RDS in preterm infants. Specifically, the study aimed to evaluate and compare short- and intermediate-term clinical outcomes, including rates of morbidity and mortality, associated with each surfactant. 

## 2. Material and Methods

### 2.1. Study Design and Patient Selection

This prospective cohort study included preterm infants (25 ^0⁄7^–32 ^6⁄7^ weeks gestation) admitted to a Level III NICU between January 2023 and March 2024, who required surfactant therapy within the first 48 h of life. Infants with major congenital anomalies, outborn status, or incomplete data were excluded. In our unit, many infants are born at less than 25 weeks of gestation without adequate antenatal follow-up. Therefore, to avoid further confounding from insufficient antenatal steroid administration or asphyxiated infants, those born before 25 weeks of gestation were not included in the analyses.

Surfactant choice depended on hospital availability: poractant alfa was available from January to November 2023, and calfactant from December 2023 to March 2024. There were no changes in clinical practices during the study period.

Surfactant administration followed the European Consensus Guidelines on the Management of Respiratory Distress Syndrome [2]. Infants <30 weeks of gestation who were intubated in the delivery room received endotracheal surfactant immediately. Non-intubated infants received surfactant via Less Invasive Surfactant Administration (LISA) or Intubation-Surfactant-Extubation (INSURE) methods. FiO_2_ ≥30% indicated the need for surfactant, with additional doses given within 6–12 h if required.

Poractant alfa was administered at 2.5 mL/kg (200 mg/kg) initially, followed by 1.25 mL/kg (100 mg/kg). Calfactant was administered at 3 mL/kg (105 mg/kg) for both initial and subsequent doses. All infants with RDS born before 32 weeks of gestation received caffeine therapy per unit protocol, consisting of a 20 mg/kg loading dose and a 10 mg/kg/day maintenance dose until 34 weeks of gestation.

### 2.2. Primary and Secondary Outcomes

The primary outcomes were moderate-to-severe BPD and/or mortality.

Bronchopulmonary dysplasia was defined as a persistent oxygen requirement at 36 weeks postmenstrual age in a preterm infant who had required supplemental oxygen for at least 28 days. Infants who required non-invasive respiratory support beyond the mild level at 36 weeks PMA—for example, nasal cannula > 2 L/min, noninvasive positive airway pressure (such as CPAP or NIPPV), or invasive mechanical ventilation—were classified as having moderate to severe BPD [8]. 

Secondary outcomes were extubation success and the need for repeated surfactant dosing. Infants were extubated and transitioned to non-invasive positive pressure ventilation (NIPPV) if they demonstrated adequate spontaneous respiration, required FiO_2_ < 30%, and maintained a mean airway pressure (MAP) < 8 cmH_2_O. Initial NIPPV settings were established with a positive inspiratory pressure (PIP) set 2 cmH_2_O above the last PIP value on invasive mechanical ventilation, a positive end-expiratory pressure (PEEP) of 6 cmH_2_O, FiO_2_ 5–10% above baseline, a respiratory rate of 45 breaths per minute, and an inspiration time of 0.4–0.5 s. Extubation failure was defined within one week following extubation by the presence of any of the following criteria: FiO_2_ > 40%, venous blood gas pH < 7.20, PCO_2_ > 60 mmHg, or apnea requiring positive pressure ventilation and reintubation.

Intubation rates were compared between surfactant groups among infants who received surfactant via the LISA or INSURE techniques. Among the less invasive methods, LISA was used as the primary approach. ENSURE was performed in only 3 infants, based on the clinician’s decision. No strict criteria were established.

Others secondary outcomes included air leak (pneumothorax), hemodynamically significant patent ductus arteriosus (PDA), severe intraventricular hemorrhage (IVH, grade ≥2) [9], necrotizing enterocolitis (NEC, stage ≥2) [10], moderate-to-severe BPD, retinopathy of prematurity (ROP) requiring treatment, respiratory support duration, hospitalization length, and mortality.

A hemodynamically significant patent ductus arteriosus (HsPDA) was diagnosed via echocardiography, indicated by high-volume ductal flow, typically performed between 48 and 72 h after birth. HsPDA was defined echocardiographically as a moderate-to-large transductal diameter (typically >1.5 mm) with a non-restrictive or pulsatile left-to-right shunt, accompanied by parameters indicating pulmonary overcirculation (e.g., increased left atrium-to-aortic root [LA/Ao] ratio, shortened isovolumic relaxation time, or elevated E:A ratio) and/or evidence of systemic hypoperfusion, such as absent or reversed diastolic flow in the descending aorta, celiac trunk, or middle cerebral artery [11].

A cost-effectiveness analysis was conducted using current local market prices, converted to US dollars at the prevailing exchange rate. Poractant alfa (1.5–3 mL) and calfactant (3–6 mL) were supplied in two vial sizes. To minimize drug waste, the vial size was selected to reduce the number of vials required per patient, and cost calculations were adjusted accordingly.

Between January 2023 and March 2024, the 1.5 cc preparation of poractant alfa was priced at $160, and the 3 cc preparation at $325. During the same period, the 3 cc preparation of calfactant was $350, while the 6 cc preparation was $606. The per-patient cost was calculated as the number of vials used × vials per patient. In our study, surfactant administration was performed in accordance with the European RDS guideline recommendations and was applied similarly in both periods. Written informed consent was obtained from the parents. Ethical approval was obtained from the local Clinical Research Ethics Committee (Date: 8 October 2021; no: 914).

### 2.3. Statistical Analysis

Data were analyzed using IBM SPSS Statistics for Windows, Version 26.0 (IBM Corp., Armonk, NY, USA; [https://www.ibm.com/products/spss-statistics]. Normality of continuous variables was assessed both visually (histograms and Q-Q plots) and analytically (Kolmogorov–Smirnov and Shapiro–Wilk tests). Normally distributed data were expressed as mean ± standard deviation and compared using Student’s *t*-test. Non-normally distributed data were expressed as median (minimum–maximum) and compared using the Mann–Whitney U test. Categorical variables were analyzed using the Chi-square or Fisher’s exact test, as appropriate. Logistic and linear regression analyses were performed, adjusted for birth weight and gestational age. Statistical significance was set at *p* < 0.05.

## 3. Results

Between January 2023 and March 2024, a total of 544 preterm infants (<32 weeks’ gestation) requiring surfactant therapy within the first 48 h of life were born in our Level III NICU. Of these, 374 were born during the period when poractant alfa was in use (January–November 2023), and 170 during the period when calfactant was used (December 2023–March 2024). After applying exclusion criteria (major congenital anomalies, gestational age <25 weeks, outborn transfers, incomplete data, and lack of parental consent), 215 infants were eligible for inclusion. Among them, 137 received poractant alfa and 78 received calfactant. A total of 111 infants were admitted from the delivery room already intubated, while 114 infants were initially managed with nasal CPAP and subsequently either intubated and placed on mechanical ventilation (51 infants) or given surfactant via the LISA/ENSURE method and continued on non-invasive ventilation. Overall, 53 infants (24.7%) received their first surfactant dose via less invasive methods (LISA or INSURE). The proportion of infants managed with these techniques was significantly higher in the poractant group compared to the calfactant group (32.8% vs. 10.3%, *p* < 0.01). Antenatal steroid administration was observed more frequently in the calfactant group (52.6% vs. 44.5%), although the difference did not reach statistical significance (*p* = 0.36). Other perinatal variables, including mode of delivery, sex distribution, and maternal comorbidities, were comparable between the two groups. Detailed demographic and perinatal characteristics are summarized in Table 1.

Evaluation of clinical outcomes demonstrated that a hemodynamically significant PDA was identified in 42.3% of infants in the poractant group and 55.1% in the calfactant group, representing a statistically significant difference (*p* = 0.048). No significant intergroup differences were observed with respect to other complications, duration of hospitalization, or overall mortality (Table 2).

Among infants who received surfactant via LISA/INSURE, 2 (4.4%) in the poractant group and 4 (50%) in the calfactant group required intubation within the first 72 h of life (*p* = 0.03). In total, 95 infants in the poractant group and 74 in the calfactant group remained intubated. Reintubation was required in 36 infants (38%) in the poractant group and 32 infants (46%) in the calfactant group, with no significant difference between groups.

The incidence of bronchopulmonary dysplasia was 35.2% (*n* = 38) in the poractant group and 51.5% (*n* = 34) in the calfactant group, which did not reach statistical significance (*p* = 0.73). Mortality rates were also comparable between groups (26.9% [*n* = 29] vs. 25.7% [*n* = 17], *p* = 0.48). Data regarding short- and mid-term morbidity as well as short-, mid-, and long-term mortality are presented in Table 3. There were no significant differences between groups in the incidence of moderate-to-severe BPD (32.8% vs. 34.6%, *p* = 0.18) or in the composite outcome of BPD or mortality (45.3% vs. 51.3%, *p* = 0.12). Although invasive mechanical ventilation on days 3 and 5 was more frequent in the calfactant group (88.5% vs. 46%), the difference was not statistically significant (*p* = 0.27). Durations of non-invasive ventilation (4 vs. 5.5 days, *p* = 0.32), mechanical ventilation (10 vs. 5 days, *p* = 0.71), total respiratory support (31 vs. 32 days, *p* = 0.87), and hospitalization (51 days in both groups, *p* = 0.25) were also comparable (Table 3). Mortality rates were similar (21.2% vs. 24.4%, *p* = 0.13). Early mortality (<3 days) was more frequent in the poractant group (34.5% vs. 23.5%), but not significant (*p* = 0.16). Mortality between days 3–7, 8–27, and >28 days showed no significant differences between groups (Table 4).

Surfactant administration parameters are summarized in Table 5. The requirement for a single surfactant dose was 62% in the poractant alfa group, which was higher than in the calfactant group (*p* = 0.021). The requirement for a second dose of surfactant was higher in the calfactant group (*p* = 0.027), whereas the need for three or more doses was similar in both groups.

A significant difference was further noted in the cost analysis. The median surfactant cost per patient was lower in the poractant group compared with the calfactant group (214.2 USD [range 214.2–856.9] vs. 350 USD [range 198–1396], *p* = 0.02). The timing of the second and third surfactant doses did not differ significantly between groups (*p* = 0.72) (Table 5).

After adjustment for birth weight and gestational age, no statistically significant differences were observed between poractant alfa and calfactant in terms of PDA, mortality, BPD, or the combined outcome of BPD/mortality. Similarly, no significant differences were found regarding the duration of invasive and non-invasive respiratory support or length of hospital stay. However, a higher rate of LISA/ENSURE therapy was observed in the poractant alfa group (Table 6).

## 4. Discussion

Respiratory distress syndrome (RDS) remains one of the leading causes of morbidity and mortality in preterm infants, and surfactant replacement therapy constitutes a cornerstone in its management. Over the past decades, the introduction of natural surfactants derived from animal lungs has significantly improved survival rates and respiratory outcomes. Among these, poractant alfa (porcine-derived) and calfactant (bovine-derived) are widely used in neonatal intensive care units (NICUs) worldwide. However, their comparative clinical effectiveness, safety profiles, and pharmacoeconomic outcomes continue to be subjects of ongoing debate [12,13,14,15,16,17]. Our study contributes further evidence by comparing the clinical, perinatal, and economic outcomes of poractant alfa and calfactant in preterm infants treated for RDS.

In this study, we compared the clinical, perinatal, and pharmacoeconomic outcomes of two natural surfactants—poractant alfa and calfactant. Our findings indicate that poractant alfa is associated with several advantages, including a higher initial dose administration, lower surfactant cost, and a reduced need for a second surfactant dose. However, no significant differences were observed between groups in terms of mortality, moderate-to-severe bronchopulmonary dysplasia (BPD), or short- and mid-term respiratory morbidities. Furthermore, when adjusted for gestational age and birth weight, the incidence of hsPDA was comparable between the two groups.

The mean gestational ages of infants in our study were 27.2 and 27.7 weeks. Although not statistically significant, infants in the poractant alfa group were more frequently born at 25–26 weeks, while those in the calfactant group were more often born at 29 weeks. Previous studies have reported a wide range of gestational ages. In the randomized controlled trial by Ramanathan et al., the mean gestational age was 28.8 and 28.7 weeks. That study, which compared two dosages of poractant alfa and beractant, found no significant differences regarding air leak syndromes, PDA, NEC, severe IVH, pulmonary hemorrhage, or the need for respiratory support [18].

Trembath et al. analyzed data from 51,282 infants treated in 322 NICUs in the US between 2012 and 2013, comparing outcomes associated with beractant, calfactant, and poractant alfa. The mean gestational age in that cohort was 30 weeks, and the mean birth weight was 1435 gr [15]. As noted by Branagan et al., most previous studies have included infants born between 27 and 30 weeks [12]. In contrast, our cohort had a mean gestational age of 27 weeks, which is slightly lower than that of previous studies.

Consistent with our findings, many previous studies have not demonstrated significant differences in mortality between natural surfactant preparations [13,14]. A randomized trial reported that 200 mg/kg poractant alfa reduced mortality compared to both 100 mg/kg poractant alfa and 100 mg/kg beractant, particularly among infants weighing 500–749 g, although this benefit was not observed in larger infants [18]. Similarly, Kadıoğlu et al. found no differences in total, early, or late postnatal mortality when comparing poractant alfa and beractant [14]. In Trembath’s study, nearly half of the cohort consisted of moderate-to-late preterm infants, and no differences were found among the three surfactants in terms of air leak, mortality, or BPD/death [15].

In our cohort, early mortality (0–7 days) was slightly higher than previously reported rates. The main contributing factors included severe RDS, unmonitored pregnancies, early and late neonatal sepsis, and pulmonary hemorrhage. In contrast, mortality beyond day seven was consistent with previous reports. Park et al. demonstrated that causes of death in infants born at 23–26 weeks vary with postnatal age: within the first 24 h, air leak, pulmonary hypoplasia, and IVH; between days 2–7, sepsis, IVH, and pulmonary hemorrhage; and between days 8–28, NEC, sepsis, pulmonary hemorrhage, air leak syndromes, and acute renal failure [19]. These findings are consistent with our observations.

On the other hand, Bui et al. found no significant differences between poractant alfa and calfactant in terms of mechanical ventilation need on day 3, BPD incidence, length of hospital stay, or mortality [3]. Similarly, Yılmaz et al. compared three natural surfactants and found no significant differences regarding hemodynamically significant PDA, BPD, NEC ≥ stage II, severe IVH, air leak, duration of ventilation, oxygen dependency, time to full enteral feeding, or length of hospital stay. Interestingly, they reported significantly lower rates of sepsis and mortality in the calfactant group [16]. Lane et al., using the Pediatrix database (1997–2017), compared three natural surfactants and found no overall differences in efficacy or safety but reported subgroup differences in safety between poractant alfa and beractant in infants born at 33–36 weeks. No significant differences were found between poractant alfa and calfactant [17].

Despite including smaller and more immature infants, our study did not find significant differences in the incidence of moderate-to-severe BPD, NEC, or severe IVH. While hsPDA was more common in the calfactant group, PDA ligation rates were similar. After adjustment for gestational age, hsPDA incidence was comparable between groups. Considering the lower gestational ages in the poractant alfa group, a higher hsPDA rate would have been expected. Fuji et al. [20] reported lower rates of hsPDA requiring treatment in the poractant alfa group compared to beractant. Poractant alfa, a porcine-derived extract, has a higher phospholipid content and contains the hydrophobic surfactant proteins SP-B and SP-C, which facilitate the rapid spreading and stabilization of the alveolar film. In contrast, calf-derived Calfactant has a lower phospholipid content and higher SP-A activity, which may be considered immunomodulatory in immature lungs. The higher phospholipid density and SP-B/SP-C richness of Poractant alfa may be associated with the faster oxygenation and resorptive unit structure reported by Boskabadi et al. [21]. The higher initial dose (e.g., 200 mg/kg) allows for faster distribution within the alveoli, improving oxygenation and reducing pulmonary vascular resistance more rapidly, thereby limiting the hemodynamic impact of ductal shunting. Additionally, the smaller fluid volume required for poractant alfa administration may reduce fluid overload and minimize pulmonary circulatory effects. Early administration of surfactant may also improve ventilation and oxygenation, reducing the significance of PDA and the need for additional treatment [20,22]. Nevertheless, large comparative and national database studies have generally reported similar hsPDA rates among different surfactants. Although transient circulatory changes after surfactant therapy have been observed, no clear superiority has been established [8,16,23].

The literature generally reports similar results regarding the duration and need for early invasive mechanical ventilation across surfactants, with small-sample studies often lacking statistical significance. Systematic reviews also fail to demonstrate any definitive superiority [24]. In our study, durations of both invasive and non-invasive ventilation were comparable between groups.

Time-related outcomes are typically similar among natural surfactants, and national data from Turkey comparing three surfactants support comparable durations and oxygen dependency. Both multicenter comparative and single-center retrospective studies have reported no significant differences in late-onset sepsis, which aligns with our findings [16,24].

Large meta-analyses have shown that LISA and INSURE techniques reduce the need for mechanical ventilation and the combined incidence of BPD or death, with these benefits attributed mainly to the method rather than the surfactant type. Poractant alfa, due to its smaller volume per dose (200 mg/kg = 2.5 mL/kg), may facilitate LISA/INSURE procedures, whereas calfactant requires a larger volume (105 mg/kg = 3 mL/kg) [2]. This may partly explain the higher frequency of LISA/INSURE use in the poractant alfa group in our cohort. Our study also found that infants receiving LISA/ENSURE in the calfactant group required more frequent intubation. However, the number of patients who received surfactant via LISA/ENSURE in the calfactant group was relatively lower.Although there are not many comparative studies in the literature, Zamal et al. reported that the ≤72-h post-LISA intubation rates were identical between beractant and poractant, at 3.3% in both groups [25].

Although the total surfactant dose per infant was significantly higher in the poractant alfa group (*p* < 0.01), the proportion of infants treated with a single dose was higher and the need for a second dose was lower in this group. The success rate of single-dose surfactant therapy varies across centers and studies. In a large, prospective single-center study involving 415 neonates, the single-dose use of poractant alfa was reported at 37% [26]. In a multicenter study of 2168 infants comparing 200 mg/kg to 100 mg/kg poractant alfa, only 31% of those receiving the higher dose required only one dose [27]. Conversely, Jeon et al. reported high single-dose usage rates for poractant alfa (77%), calfactant (83%), and beractant (83%) in their sequential cohort study [28]. In our study, the rate of single-dose administration was 62% in the poractant alfa group and 44% in the calfactant group (*p* = 0.021). The need for a second dose was significantly lower in the poractant alfa group (*p* = 0.027). Despite being used in infants with lower gestational age, poractant alfa demonstrated improved efficacy and was associated with more favorable outcomes. However, in a multicenter observational study by Trembath and colleagues comparing three surfactants, no statistically significant difference was found between groups in the need for repeat doses [15]. 

Total mg/kg dosing was consistent with product recommendations (poractant alfa: 200 mg/kg; calfactant: 105 mg/kg). Randomized evidence supports that 200 mg/kg poractant alfa reduces mortality compared to 100 mg/kg poractant alfa and beractant in certain subgroups [2,6]. However, in our study, high-dose administration was not associated with significant clinical differences. Real-world data also show no substantial variation in repeat doses or dosing intervals [16].

In our cost analysis, the average per-infant surfactant cost was $214 for poractant alfa and $350 for calfactant (*p* = 0.02). These findings differ from those of Bui et al., who reported similar retreatment rates but higher total dose requirements in the calfactant group [3]. Zayek et al. also reported slightly higher per-infant dosing requirements for calfactant compared to poractant alfa [29]. Importantly, poractant alfa’s smaller administration volume allows for faster delivery, potentially reducing reflux and improving cost-effectiveness. However, Bui et al. calculated higher annual costs for poractant alfa, and Zayek et al. reported higher total and per-infant costs for poractant alfa [3,23]. We attribute our cost findings primarily to local drug pricing policies.

The main limitation of our study is its non-randomized design; surfactant selection was based on hospital availability rather than random allocation, introducing the possibility of selection bias. Moreover, long-term neurodevelopmental outcomes were not assessed. Differences in LISA/INSURE application between groups may have influenced outcomes. Although surfactant administration adhered to current guidelines, the use of different surfactants during separate time periods may have led to variations in clinical practice, potentially affecting results. Another limitation is that we did not perform adjustments for multiple comparisons. These limitations restrict the generalizability of our findings and highlight the need for larger, multicenter randomized studies.

## 5. Conclusions

In our study, no significant differences were observed between groups in terms of clinical morbidity and mortality. However, poractant alfa may be a more advantageous option, given its lower requirement for repeat dosing and lower cost.

## Figures and Tables

**Table 1:** Perinatal Characteristics of Patients Receiving Poractant Alfa and Calfactant.

Characteristics	PoractantAlfa Group (*n* = 137)	Calfactant Group (*n* = 78)	*p*-Value
Birth weight, g (mean ± SD)	1012 ± 222	1066 ± 232	0.096
Gestational age, weeks (mean ± SD)	27.2 ± 1.8	27.7 ± 1.5	0.067
25 weeks, *n* (%)	24 (17.5%)	6 (7.7%)	0.033
26 weeks, *n* (%)	35 (25.5%)	11 (14.1%)	0.034
27 weeks, *n* (%)	23 (16.8%)	20 (25.6%)	0.084
28 weeks, *n* (%)	25 (18.2%)	17 (21.8%)	0.32
29 weeks, *n* (%)	12 (8.8%)	15 (19.2%)	0.024
30 weeks, *n* (%)	11 (8%)	7 (9%)	0.42
31 weeks, *n* (%)	8 (5.8%)	2 (2.6%)	0.23
Antenatal steroids, *n* (%)	61 (44.5%)	41 (52.6%)	0.36
Male sex, *n* (%)	78 (58.7%)	42 (49%)	0.22
Cesarean delivery, *n* (%)	120 (87.6%)	70 (89.7%)	0.82
Multiple birth, *n* (%)	31 (22.6%)	26 (33.3%)	0.51
Maternal preeclampsia, *n* (%)	13 (9.5%)	7 (5.1%)	0.47
Maternal diabetes, *n* (%)	2 (1.5%)	2 (2.6%)	1.00
PPROM, *n* (%)	12 (8.7%)	9 (11.5%)	1.00
EMR, *n* (%)	24 (17.5%)	20 (25.6%)	0.86
Chorioamnionitis, *n* (%)	8 (5.8%)	3 (3.8%)	0.39
Neonatal resuscitation at delivery	80 (58.4%)	59 (75.6%)	0.067
Apgar score at 1 min, median (min-max)	5 (2–8)	5 (1–8)	0.10
Apgar score at 5 min, median (min-max)	7 (4–8)	7 (2–9)	0.94
Apgar score at 5 min <7, *n* (%)	69 (50.4%)	30(38.5%)	0.11
pH	7.28± 0.10	7.29± 0.11	0.77
BE	−6.2± 4.2	−5.6± 4.5	0.36
LISA/INSURE	45 (32.8%)	8 (10.3%)	<0.01

PPROM: Preterm premature rupture of membranes; EMR: Early membrane rupture; LISA: Less invasive surfactant administration; INSURE: Intubation–surfactant–extubation.

**Table 2:** Clinical Characteristics of Patients Receiving Poractant Alfa and Calfactant.

Characteristic	PoractantAlfa Group (*n* = 137)	Calfactant Group (*n* = 78)	*p*-Value
Pulmonary hemorrhage, *n* (%)	15 (10.9%)	10 (12.8%)	0.67
HsPDA, *n* (%)	58 (42.3%)	43 (55.1%)	0.048
PDA ligation, *n* (%)	5 (2.8%)	3 (4.5%)	0.605
Pneumothorax, *n* (%)	6 (4.4%)	2 (2.6%)	0.208
Severe IVH (Grade III–IV), *n* (%)	24 (17.5%)	8 (10.3%)	0.106
Periventricular leukomalacia (PVL), *n* (%)	19 (13.9%)	10 (12.8%)	0.502
Extubation failure, *n* (%)	33 (30.5%)	27 (41%)	0.35
Necrotizing enterocolitis, *n* (%)	8 (5.8%)	5 (6.4%)	0.155
Retinopathy of prematurity, *n* (%)	9 (6.6%)	3 (3.8%)	0.147
Early-onset sepsis, *n* (%)	49 (37.8%)	23 (29.5%)	0.216
Late-onset sepsis, *n* (%)	103 (75.2%)	61 (78.2%)	0.37
Duration of hospitalization (days), median (min-max)	47(4–274)	48 (4–183)	0.31
Mortality, *n* (%)	29 (21.2%)	19 (24.4%)	0.13

Values are presented as median (range) for continuous variables and number (percentage) for categorical variables. HsPDA: Hemodynamically significant patent ductus arteriosus; IVH: Intraventricular hemorrhage; PVL: Periventricular leukomalacia.

**Table 3:** Clinical and Respiratory Parameters of Patients Receiving Poractant Alfa and Calfactant.

Parameter	PoractantAlfa Group (*n* = 137)	Calfactant Group (*n* = 78)	*p*-Value
Bronchopulmonary dysplasia (moderate–severe), *n* (%)	45 (32.8%)	27 (34.6%)	0.18
BPD or mortality, *n* (%)	62 (45.3%)	40 (51.3%)	0.12
Invasive mechanical ventilation on day 3, *n* (%)	63 (46%)	69 (88.5%)	0.27
Invasive mechanical ventilation on day 5, *n* (%)	63 (46%)	69 (88.5%)	0.27
Duration of non-invasive ventilation (days), median (min-max)	4 (0–42)	5.5 (0–28)	0.32
Duration of mechanical ventilation (days), median (min-max)	10 (0–140)	5 (0–53)	0.71
Duration of total respiratory support (days), median (min-max)	31 (1–274)	32 (1–95)	0.87
Respiratory support requirement on day 28, *n* (%)	46 (33.6)	44 (56.4)	0.29
Duration of hospitalization (days), mean ± SD	51.0 ± 3.9	51.0 ± 2.6	0.25

BPD: Bronchopulmonary dysplasia.

**Table 4:** Timing of Mortality in Patients Receiving Poractant Alfa and Calfactant.

Parameter	PoractantAlfa Group (*n* = 137)	Calfactant Group (*n* = 78)	*p*-Value
0–3 days, *n* (% of deaths)	10 (34.5%)	4 (21%)	0.16
3–7 days, *n* (% of deaths)	5 (17.2%)	2 (10.6%)	0.31
8–27 days, *n* (% of deaths)	9 (31%)	7 (36.7%)	0.56
>28 days, *n* (% of deaths)	5 (17.2%)	6 (31.6%)	0.39
Total mortality, *n* (%)	29 (21.2%)	19 (24.4%)	0.13

**Table 5:** Surfactant Administration Parameters in Patients Receiving Poractant Alfa and Calfactant.

Parameter	PoractantAlfa Group (*n* = 137)	Calfactant Group (*n* = 78)	*p*-Value
Requirement for single of surfactant, *n* (%)	85 (62)	34 (44)	0.021
Requirement for 2 doses of surfactant, *n* (%)	35 (25.5)	32 (41)	0.027
Requirement for 3 or more doses of surfactant, *n* (%)	17 (12.4)	12 (8.6)	0.334
Total surfactant dose (mg/kg, median, min-max)	200 (100–500)	100 (105–420)	<0.001
Time of second dose surfactant administration, hour median (min-max)	10 (4–48)	12 (4–46)	0.15
Time of third dose surfactant administration, median (min-max)	12 (8–96)	24 (8–96)	0.72
Surfactant cost per patient, ($), median (min-max)	214.2 (214.2–856.9)	350 (198–1396)	0.02

**Table 6:** Adjusted Regression Analysis of Clinical Outcomes in Preterm Infants Treated with Poractant Alfa versus Calfactant.

Outcome	Model	Adjusted Effect	95% CI Lower	95% CI Upper	*p*-Value
HsPDA	Logistic (OR)	1.873	0.615	5.711	0.269
Mortality	Logistic (OR)	1.339	0.617	2.908	0.460
BPD or Mortality	Logistic (OR)	0.828	0.433	1.583	0.568
BPD	Logistic (OR)	0.757	0.379	1.511	0.429
Duration of mechanical ventilation (days)	Logistic (OR)	0.852	0.701	1.035	0.107
Duration of non-invasive ventilation (days)	Logistic (OR)	0.955	0.788	1.157	0.637
Duration of hospitalization (days)	Logistic (OR)	0.999	0.975	1.035	0.968
LISA/ENSURE	Logistic (OR)	0.212	0.093	0.485	0.000

## Data Availability

Datasets analyzed or generated during this study are presented in the current study.
